# Investigating prolonged social withdrawal behaviour as a risk factor for self-harm and suicidal behaviours

**DOI:** 10.1192/bjo.2021.47

**Published:** 2021-04-30

**Authors:** Shimin Zhu, Paul H. Lee, Paul W. C. Wong

**Affiliations:** Department of Applied Social Sciences, The Hong Kong Polytechnic University, Hong Kong; Department of Health Sciences, University of Leicester, UK; Department of Social Work and Social Administration, The University of Hong Kong, Hong Kong

**Keywords:** Self-harm, suicidal behaviour, *hikikomori*, prolonged social withdrawal behaviour, China

## Abstract

**Background:**

Self-harm and suicidal behaviour are recognised as public health concerns. Prolonged social withdrawal behaviour, or *hikikomori*, is reported as a risk factor for suicidal behaviour.

**Aims:**

To examine the occurrence and additional risk of prolonged social withdrawal behaviour on self-harm and suicidal behaviour among Chinese university students.

**Method:**

A cross-sectional online survey was conducted with three universities in southern China. A two-stage random sampling was adopted for recruitment, with students in different years of study, in different departments of each participating university. Hierarchical logistic regression analyses were conducted to investigate the sociodemographic and psychological correlates of self-harm and suicidal behaviours among male and female participants with *hikikomori* status.

**Results:**

Of the students who completed the online survey, 1735 (72.23%) were included in the analysis; 11.5% (*n* = 200) reported self-harm behaviour and 11.8% (*n* = 204) reported suicidal behaviours in the past 12 months. Men showed a higher prevalence rate of self-harm than women (14.7% *v.* 10.8%, *P* = 0.048), but a similar rate of suicidal behaviours (11.9% *v.* 11.3%, *P* = 0.78). The overall prevalence rate of social withdrawal behaviour was 3.2% (7.0% for men and 2.3% for women, *P* < 0.001). Prolonged social withdrawal behaviour status was significantly associated with self-harm (odds ratio 2.00, 95% CI 1.22–3.29) and suicidal behaviour (odds ratio 2.35, 95% CI 1.45–3.81). However, the associations became statistically insignificant after adjustment for psychological factors in the final models in the logistic regression analyses.

**Conclusions:**

Prolonged social withdrawal behaviour appears to be associated with self-harm and suicidal behaviour, but psychological factors have stronger links with suicidality.

Suicide is highly related to poor mental health conditions and is the second most common cause of death in 15- to 24-year-olds worldwide.^1,2^ It is estimated that between 15 and 30 million individuals engaged in self-harm behaviour in 2020.^3^ The most recent findings of the Global School-Based Health Survey, conducted in 83 countries, found that young people aged 12–15 years had thought about (16.5%), planned for (16.5%) and attempted suicide (16.4%) in the past 12 months.^4^ Self-harm and suicidal behaviour have a considerable risk of future suicide, especially among males, older adolescents and those who repeatedly self-harm,^5^ and such risk could persist over several years.^6^ Suicide and self-harm also lead to extensive societal and economic cost, including years of life lost, years of productive life lost and present economic value of lost productivity. For instance, it was estimated that the 6912 young people who died of suicide in Norway, Australia, Switzerland, Germany, Denmark, Singapore, the Netherlands, Ireland, Canada and the USA in 2014 led to 406 730 years of life lost, at a cost of US$5.53 billion, with the average cost of each suicide estimated at US$802 939.^7^

## Prolonged social withdrawal and suicidality

Prolonged social withdrawal behaviour among youth, or *hikikomori*, is gaining attention on its own and in terms of its relationship with self-harm and suicidal behaviour. Young people who experience a prolonged period of social withdrawal for 6 months or longer, with little to no social interaction, but who do not have any severe psychiatric disorders, have been referred to as *hikikomori*.^8^ *Hikikomori* was initially thought to be a Japanese phenomenon because of Japan's specific cultural, historical and economic circumstances, but there has been an increase in the identification of similar cases in other Asian countries, including China, Hong Kong and Taiwan, as well as non-Asian countries, including Italy and Spain.^9^ Prolonged social withdrawal behaviour negatively influences not only an individual's mental and social well-being, but also wider education and workforce participation and stability, especially during the COVID-19 period.^10^ The relationship between prolonged social withdrawal behaviour and suicide is yet to be adequately elucidated, but several cases of persons with prolonged social withdrawal behaviour who have attempted and completed suicide have been reported in case studies.^8^ In a prevalence study on prolonged social withdrawal behaviour among people aged 12–29 years in Hong Kong,^11^ those who had been withdrawn for more than 3 months were 2.3 times more likely to engage in self-harm than non-withdrawn young people. A recent secondary data analysis study, using the data from a survey of 5000 people aged 15–39 years in Japan, found that the participants with one or more suicide risk factors had 2.8 times greater chance of being a person with prolonged social withdrawal behaviour.^12^ Since prolonged social withdrawal behaviour and suicidal behaviour may be engaged as ways of detaching oneself from negative life events and society in general, addressing the associations between self-harm behaviour and prolonged social withdrawal behaviour will help to prevent two general intentional self-harming behaviours among young people.

## Mental well-being of university students in China

In addition, previous studies have reported that the epidemiologic characteristics of both self-harm and prolonged social withdrawal behaviour have differences based on gender. In general, females are more likely to engage in self-harm behaviour^13^ and males are more likely to engage in prolonged social withdrawal behaviour.^14^ In China, suicide is the leading cause of death for young people,^15,16^ and previous studies have suggested that positive psychological factors, such as hope, purpose of life, flourishing and belief in change, are factors that protect against self-harm and suicidal behaviour among university students.^17,18^ Conversely, mental health symptoms are closely related to social withdrawal, self-harm and suicidal behaviours.^19^ Furthermore, prolonged social withdrawal among young people is increasing in China.^20^ We are unaware of any surveys that examine the association between self-harm and prolonged social withdrawal behaviour among young people in China. Therefore, this study aimed to investigate whether prolonged social withdrawal behaviour confers additional risk for self-harm or suicidal behaviours related to the poor psychological status of university students in China. Since gender difference plays a significant role in the understanding of self-harm and social withdrawal behaviours, we also examined whether the potential gender differences are present within the context of this association. We hypothesised that prolonged social withdrawal behaviour would have an additional impact on, and contribute to, the self-harm or suicidal behaviour related to the poor psychological status of university students.

## Method

### Recruitment and sampling

We sent invitations to the student support centres of eight medium-sized universities in a large city in southern China in 2018. Three universities, each with around 10 000 students, indicated interest in participation. The first author then introduced the study and data collection method to at least three members of the student support centre at each of the universities. To recruit participants as randomly as possible and ensure their voluntariness and anonymity, the data collection process included three steps. First, the teachers randomly selected five departments in their university and then randomly selected students in two years of study in each department. Second, the teachers sent an information sheet and the survey link to the class groups through WeChat. It was clearly stated that students could volunteer to participate and there would be no negative consequence if they did not participate or withdrew from the survey. The teachers would not know who had participated or how participants had answered the survey questions. Third, the teacher sent a reminder after 2 days. About 1 week after the first invitation, the teacher repeated this random selection process and invited students from other departments. The whole survey was rolled out for 2 weeks, and closed when the targeted sample size was met. The survey was completed in the middle of the semester, to minimise the influence of adaptation to a new semester and the academic stress induced by examinations toward the end of the semester. The Hong Kong Polytechnic University institutional review board approved all the procedures (approval number HSEARS20180521002), and all participants provided web-based consent.

To enhance the quality of the data collected through the online survey, three strategies were adopted. First, students received the survey link with an instruction, ‘If you answer the survey carefully, you will receive an immediate individual report of your mental health status and can receive a WeChat cash reward (US$2) after a quality check’. Second, a pilot study of 30 university students was conducted, and found that the mean time to finish the survey was 627 seconds (s.d. 290 seconds). Accordingly, participants with a response time that was 1 s.d. below (≤337 seconds) this were excluded from the analysis. Finally, five attention-checking items were used to screen careless responding (e.g. ‘Please answer Choice 2 to ensure you are paying attention’). Only those who answered all five attention-checking items correctly were included in the analysis.^21^

### Instruments

The measures consisted of sociodemographic information, self-harm, suicidal behaviour and social withdrawal behaviour, and were used to examine the association between prolonged social withdrawal behaviour and self-harm and suicidal behaviour. We also included the psychological protective factors and mental health-related risk factors, which were adopted in recent research of suicidality among Chinese university students,^22,23^ to further examine the association after controlling relevant protective and risks factors.

#### Background information of the participants

Sociodemographic information, including gender, age, the university the participant attended, major, year of study in the four-year curriculum, how many years they have lived in the city, grade point average (GPA), type of hometown area (city, town or rural) and parents’ education level, were collected.

#### Self-harm, suicidal behaviour and social withdrawal behaviour

##### Self-harm

Self-harm was defined as self-injurious behaviour without suicidal intent. Respondents were asked to respond with a dichotomous yes/no to the following question: ‘Some youth intentionally injure themselves or induce pain. This act aims to alleviate psychological distress but not to kill themselves. In the past 12 months, did you have such an experience?’ If they had experienced such an event, they were then asked which method they used to injure themselves. Six options were provided, and more than one method could be chosen, including cut or stab yourself with a needle, knife or nails (sharp instrument); use fire or hot objects to cause a burn injury (burning); punch or hit a wall or hard object (punching); reckless and risky behaviours like jumping from a moving car (reckless and risky behaviours); drug overdose and others. These questions were answered with dichotomous yes/no responses. Having engaged in any one of the six types of behaviours indicated self-harm.^24^

##### Suicidal behaviour

Respondents were asked three separate questions: ‘In the past 12 months, did you seriously consider suicide?’; ‘In the past 12 months, did you intentionally attempt suicide?’; and ‘In the past 12 months, did you attempt suicide that resulted in injury and treatment by medical personnel (doctor or nurse)?’. These questions were answered with dichotomous yes/no responses. Suicidal behaviour was defined as any endorsement of the above three questions.^24^

##### Social withdrawal behaviour

Social withdrawal behaviour was measured by the following items, which have been used in previous studies in Hong Kong,^11^ Taiwan^25^ and China.^26^ Three key questions were asked with a dichotomous yes/no answer, and if yes, for how long: ‘(1) Are you mostly spending all day at home (or dormitory)?’; ‘(2) Do you currently avoid social occasions and access to people?’ and ‘Are you distressed by either (1) or (2)?’. If any of the participants answered yes to all three questions, and for longer than 3 months, they were classified as individuals with prolonged social withdrawal behaviour.

#### Psychological and mental well-being variables

##### Hope

We used the State Hope Scale^27^ to measure the belief in participants’ capacity to initiate and sustain actions, and the belief in their capacity to generate routes. The scale comprises six items that assess the agency and pathways to reach goals, ranging from 1 (definitely false) to 8 (definitely true). The mean of all six items was taken to measure the state of hope, with a higher score meaning a stronger hopeful psychological mindset. An example item was: ‘If I should find myself in a jam, I could think of many ways to get out of it’. Cronbach's alpha for the current study was 0.895.

##### Flourishing

The flourishing scale^28^ was used to measure self-perceived flourishing and positive feeling. The scale comprises eight items related to important aspects of human functioning, including positive relationships, feelings of competence and having meaning and purpose in life. Each item was scored on a seven-point Likert scale, rating from 1 (strongly disagree) to 7 (strongly agree). A higher mean score indicates relatively higher levels of flourishing. An example item was: ‘My social relationships are supportive and rewarding’. Cronbach's alpha for the current study was 0.919.

##### Purpose in life

The four-item Purpose in Life Test-Short Form^29,30^ was used to measure participants’ self-perceived meaning and life purpose. Each item was scored on a seven-point Likert scale, from 1 (strongly disagree) to 7 (strongly agree), with the summed score ranging from 4 to 28. Higher scores suggested higher general perceived meaning of life. An example item is: ‘I have clear life goals present in my life’. Cronbach's alpha for the current study was 0.916.

##### Depression, anxiety and stress

We used the 21-item Depression Anxiety and Stress Scale (DASS-21)^31,32^ to measure participants’ emotional states of depression, anxiety and stress. The DASS-21 (*α* = 0.919) comprises three separate seven-item subscales that assess depression (*α* = 0.835), anxiety (*α* = 0.796) and stress (*α* = 0.799) symptoms, respectively. Each item was scored from 0 (did not apply to me at all) to 3 (applied to me very much or most of the time). The scores for depression, anxiety and stress were summed up and multiplied by two. Example items were: ‘I found it hard to wind down’, ‘I was aware of dryness of my mouth’ and ‘I couldn't seem to experience any positive feeling at all’.

##### Mindset of negative emotional states

The Mindset of Depression, Anxiety and Stress Scale is a 12-item scale that assesses an individual's belief of change in negative emotional states (i.e., depression, anxiety and stress).^33^ Each item was scored on a six-point Likert scale, ranging from 1 (strongly disagree) to 6 (strongly agree). By calculating the mean of scores, a higher score means a more fixed mindset of negative emotions. Sample items are: ‘When you have a certain level of depression, you really cannot do much to change it’, ‘To be honest, people cannot really change how anxious they are’ and ‘No matter how hard people try, they cannot really change the level of stress that they have’. The Cronbach's alpha for the current study was 0.942.

##### Attitude toward seeking help

The Attitude Toward Seeking Professional Help Assessment (ATSPHA)^34,35^ was used to measure participants’ views of counselling and attitudes toward seeking counselling help. The ATSCHA included 11 items (*α* = 0.719), ranging from 1 (strongly disagree) to 5 (strongly agree), and the average of the 11 items was scored as an indicator of a participant's counselling attitude. An example item was: ‘If I believed I was having a mental breakdown my first inclination would be to get professional attention’. A higher score indicates a positive attitude toward seeking help.

### Data analysis

Analysis involved all participants and was stratified by gender. Frequencies and proportions for categorical variables or mean (s.d.) for continuous variables were used to describe the characteristics of the participants. *χ*^2^-tests or independent sample *t*-tests were used to examine the association between demographic and socioeconomic characteristics, and the psychological variables on self-harm and suicidal behaviour. Hierarchical logistic regression was used to examine the association between social withdrawal status and self-harm and suicidal behaviour. Three levels of adjustment were conducted: a crude model; model 1, which was adjusted for demographic and socioeconomic characteristics; and model 2, which was adjusted for the variables listed in model 1 and psychological measures, including hope, flourishing, purpose in life, well-being, mental problems, mindset and attitude toward seeking help. Missing data were list-wise deleted. Significance level was set as *P* < 0.05. Statistical analyses were conducted with SPSS for Windows statistics, version 25.

## Results

Web-based informed consent was obtained from all 2402 participants before joining the online survey. Some students were excluded: 5 (0.21%) were not university students, 27 (1.12%) did not consent to participate and 635 (68% female, 27% male and 5% unidentified) finished the survey but their responses were filtered out by the three quality-checking strategies. Finally, 1735 (72.23%) students were included in the analysis (18.8% male). More responses from men failed to meet the quality requirement (*χ*^2^ = 25.14, *P* < 0.05) and more responses from women were included in the analysis. Participants’ mean age was 20.65 years (s.d. 1.34, range 18–25 years); 28.7% of participants were from cities, 33.7% were from townships and 37.6% were from rural areas; 27.3% of participants majored in arts or social sciences and 72.7% majored in sciences; and only 8.1% of participants were in their final year (see Table 1).

**Table 1:** Social demographic and psychological characteristics of the participants

	Female (*n* = 1408)	Male (*n* = 327)	Total (*n* = 1735)
Age, mean (s.d.)	20.66 (1.34)	20.61 (1.31)	20.65 (1.34)
Type of hometown, *n* (%)			
City	404 (28.7)	94 (28.7)	498 (28.7)
Town	470 (33.4)	114 (34.9)	584 (33.7)
Village	534 (37.9)	119 (36.4)	653 (37.6)
Time of stay, *n* (%)			
≤12 months	340 (24.1)	83 (25.4)	423 (24.4)
13–24 months	267 (19.0)	86 (26.3)	353 (20.3)
25–36 months	274 (19.5)	47 (14.4)	321 (18.5)
≥37 months	527 (37.4)	111 (33.9)	638 (36.8)
Major, *n* (%)			
Art	428 (30.4)	45 (13.8)	473 (27.3)
Science	980 (69.6)	282 (86.2)	1262 (72.7)
Year of study, *n* (%)			
Year one	432 (30.7)	114 (35.2)	546 (31.6)
Year two	382 (27.2)	124 (38.3)	506 (29.2)
Year three	462 (32.9)	75 (23.1)	537 (31.0)
Year four	130 (9.2)	11 (3.4)	141 (8.2)
Grade point average, *n* (%)			
< 2.50	88 (6.3)	58 (17.8)	146 (8.4)
2.50–2.99	279 (19.9)	116 (35.7)	395 (22.9)
3.00–3.49	531 (37.8)	97 (29.8)	628 (36.3)
3.50–3.99	418 (29.8)	41 (12.6)	459 (26.6)
≥ 4.00	87 (6.2)	13(4.0)	100 (5.8)
Self-harm^a^, *n* (%)			
No	1256 (89.2)	279 (85.3)	1535 (88.5)
Yes	152 (10.8)	48 (14.7)	200 (11.5)
Cut or stab yourself with needle, knife or nails	34 (2.4)	4 (1.2)	38 (2.2)
Use fire or hot objects to cause burn injury	3 (0.2)	2 (0.6)	5 (0.3)
Punch or hit on the wall or hard object	62 (4.4)	29 (8.9)	91 (5.2)
Reckless and risky behaviours like jumping from a moving car	9 (0.6)	5 (1.5)	14 (0.8)
Drug overdose	4 (0.3)	3 (0.9)	7 (0.4)
Other	70 (5.0)	17 (5.2)	87 (5.0)
Suicidal behaviour^b^, *n* (%)			
No	1241 (88.1)	290 (88.7)	1531 (88.2)
Yes	167 (11.9)	37 (11.3)	204 (11.8)
Suicidal thoughts	157 (11.2)	36 (11.0)	193 (11.1)
Suicide attempt	35 (2.5)	3 (0.9)	38 (2.2)
Suicidal behaviour requiring hospital assistance	2 (0.1)	1 (0.3)	3 (0.2)
Social withdrawal^c^, *n* (%)			
No	1375 (97.7)	304 (93.0)	1679 (96.8)
Yes	33 (2.3)	23 (7.0)	56 (3.2)
Hope, mean (s.d.)	4.70 (1.29)	4.80 (1.29)	4.72 (1.29)
Flourishing, mean (s.d.)	39.81 (8.23)	39.71 (8.39)	39.79 (8.26)
Purpose in life, mean (s.d.)	18.94 (4.73)	18.88 (4.93)	18.93 (4.76)
Depression, mean (s.d.)	7.21 (7.26)	7.98 (8.10)	7.35 (7.43)
Anxiety, mean (s.d.)	9.45 (7.32)	9.15 (7.76)	9.40 (7.40)
Stress, mean (s.d.)	11.74 (7.79)	11.84 (8.32)	11.76 (7.89)
Mindset, mean (s.d.)	2.92 (0.94)	2.87 (0.94)	2.91 (0.94)
Seeking help, mean (s.d.)	3.34 (0.47)	3.20 (0.52)	3.31 (0.48)

a. Self-harm was defined as self-injurious behaviour without suicide intent, measured by the six-item Non-Suicidal Self-Injury Assessment Tool (NSSI-AT). Having engaged in any one of the six types of behaviours indicates self-harm. Please refer to Supplementary Table 1a for the frequency and percentage of each item.

b. Suicidal behaviour was measured by three dichotomous (yes/no) questions about having suicidal behaviour, including suicidal thoughts, suicidal attempt and suicidal behaviour requiring hospital assistance. Suicidal behaviour was defined as any endorsement of the above three questions. Please refer to Supplementary Table 1b for the frequency and percentage of each item.

c. Social withdrawal was measured by two key items, including ‘Spend all day at home’ and ‘Avoid social occasions and access to people’.

Overall, 200 (11.5%) participants reported self-harm behaviour and 204 (11.8%) reported suicidal behaviours in the past 12 months (see Tables 2 and 3, respectively). About 11.1% of participants reported suicidal thoughts and 2.2% reported a suicide attempt; three cases reported suicidal behaviour that required hospital assistance. Specifically, male participants had higher prevalence rates of self-harm (14.7% *v.* 10.8%, *P* = 0.048), but not statistically higher suicidal behaviours (11.9% *v.* 11.3%, *P* = 0.78), than female participants. The prevalence of both self-harm (22.7%) and suicidal behaviours (16.0%) of year 3 male students were highest across gender and year of study.

**Table 2:** Descriptive statistics of self-harm, by gender

	Female			Male			Total		
	No	Yes	*P*-value	No	Yes	*P*-value	No	Yes	*P*-value
Total^a^, *n* (%)	1256 (89.2)	152 (10.8)		279 (85.3)	48 (14.7)		1535 (88.5)	200 (11.5)	
Type of hometown^b^, *n* (%)			0.053			0.299			0.152
City	353 (87.4)	51 (12.6)		78 (83.0)	16 (17.0)		431 (86.5)	67 (13.5)	
Town	413 (87.9)	57 (12.1)		102 (89.5)	12 (10.5)		515 (88.2)	69 (11.8)	
Village	490 (91.8)	44 (8.2)		99 (83.2)	20 (16.8)		589 (90.2)	64 (9.8)	
Time of stay^b^, *n* (%)			0.859			0.888			0.983
≤12 months	300 (88.2)	40 (11.8)		72 (86.7)	11 (13.3)		372 (87.9)	51 (12.1)	
13–24 months	241 (90.3)	26 (9.7)		72 (83.7)	14 (16.3)		313 (88.7)	40 (11.3)	
25–36 months	246 (89.8)	28 (10.2)		39 (83.0)	8 (17.0)		285 (88.8)	36 (11.2)	
≥37 months	469 (89.0)	58 (11.0)		96 (86.5)	15 (13.5)		565 (88.6)	73 (11.4)	
Major^b^, *n* (%)			0.146			0.014			0.053
Art	374 (87.4)	54 (12.6)		33 (73.3)	12 (26.7)		407 (86.0)	66 (14.0)	
Science	882 (90.0)	98 (10.0)		246 (87.2)	36 (12.8)		1128 (89.4)	134 (10.6)	
Year of study^b^, *n* (%)			0.320			0.036			0.548
Year one	379 (87.7)	53 (12.3)		104 (91.2)	10 (8.8)		483 (88.5)	63 (11.5)	
Year two	346 (90.6)	36 (9.4)		107 (86.3)	17 (13.7)		453 (89.5)	53 (10.5)	
Year three	417 (90.3)	45 (9.7)		58 (77.3)	17 (22.7)		475 (88.5)	62 (11.5)	
Year four	112 (86.2)	18 (13.8)		8 (72.7)	3 (27.3)		120 (85.1)	21 (14.9)	
Grade point average^b^, *n* (%)			0.786			0.317			0.277
< 2.50	78 (88.6)	10 (11.4)		46 (79.3)	12 (20.7)		124 (84.9)	22 (15.1)	
2.50–2.99	251 (90.0)	28 (10.0)		99 (85.3)	17 (14.7)		350 (88.6)	45 (11.40	
3.00–3.49	471 (88.7)	60 (11.3)		82 (84.5)	15 (15.5)		553 (88.1)	75 (11.9)	
3.50–3.99	372 (89.0)	46 (11.0)		37 (90.2)	4 (9.8)		409 (89.1)	50 (10.9)	
≥ 4.00	81 (93.1)	6 (6.9)		13 (100.0)	0 (0.0)		94 (94.0)	6 (6.0)	
Social withdrawal^b^, *n* (%)			0.051			0.005			<0.001
Yes	26 (78.8)	7 (21.2)		15 (65.2)	8 (34.8)		41 (73.2)	15 (26.8)	
No	1230 (89.5)	145 (10.5)		264 (86.8)	40 (13.2)		1494 (89.0)	185 (11.0)	
Hope^a^, mean (s.d.)	4.77 (1.26)	4.13 (1.44)	<0.001	4.90 (1.29)	4.22 (1.12)	0.001	4.79 (1.26)	4.15 (1.37)	<0.001
Flourishing^a^, mean (s.d.)	40.39 (8.02)	35.01 (8.42)	<0.001	40.41 (8.16)	35.60 (8.61)	<0.001	40.39 (8.05)	35.15 (8.45)	<0.001
Purpose in life^a^, mean (s.d.)	19.23 (4.56)	16.55 (5.40)	<0.001	19.33 (4.76)	16.29 (5.21)	<0.001	19.25 (4.59)	16.49 (5.34)	<0.001
Depression^a^, mean (s.d.)	6.51 (6.62)	12.97 (9.45)	<0.001	7.18 (7.77)	12.63 (8.50)	<0.001	6.63 (6.85)	12.89 (9.21)	<0.001
Anxiety^a^, mean (s.d.)	8.79 (6.81)	14.91 (8.96)	<0.001	8.42 (7.31)	13.42 (8.94)	0.001	8.72 (6.90)	14.55 (8.95)	<0.001
Stress^a^, mean (s.d.)	11.05 (7.39)	17.43 (8.68)	<0.001	10.98 (7.89)	16.83 (9.04)	<0.001	11.04 (7.48)	17.29 (8.75)	<0.001
Mindset^a^, mean (s.d.)	2.85 (0.91)	3.49 (1.02)	<0.001	2.81 (0.94)	3.22 (0.88)	0.005	2.85 (0.91)	3.43 (0.99)	<0.001
Seeking help^a^, mean (s.d.)	3.35 (0.46)	3.23 (0.49)	0.003	3.22 (0.51)	3.07 (0.51)	0.049	3.33 (0.47)	3.19 (0.50)	<0.001

a. *t*-test indicates a significant difference between groups of no self-harm (no) and self-harm (yes) if *P* < 0.05.

b. *χ*^2^-test indicates a significant difference between groups of no self-harm (no) and self-harm (yes) if *P* < 0.05.

**Table 3:** Descriptive statistics of suicidal behaviour, by gender

	Female			Male			Total		
	No	Yes	*P*-value	No	Yes	*P*-value	No	Yes	*P*-value
Total^a^, *n* (%)	1241 (88.1)	167 (11.9)		290 (88.7)	37 (11.3)		1531 (88.2)	204 (11.8)	
Type of hometown^b^, *n* (%)			0.314			0.856			0.403
City	348 (86.1)	56 (13.9)		84 (89.4)	10 (10.6)		432 (86.7)	66 (13.3)	
Town	420 (89.4)	50 (10.6)		102 (89.5)	12 (10.5)		522 (89.4)	62 (10.6)	
Village	473 (88.6)	61 (11.4)		104 (87.4)	15 (12.6)		577 (88.4)	76 (11.6)	
Time of stay^b^, *n* (%)			0.229			0.837			0.208
≤12 months	302 (88.8)	38 (11.2)		75 (90.4)	8 (9.6)		377 (89.1)	46 (10.9)	
13–24 months	244 (91.4)	23 (8.6)		77 (89.5)	9 (10.5)		321 (90.9)	32 (9.1)	
25–36 months	237 (86.5)	37 (13.5)		42 (89.4)	5 (10.6)		279 (86.9)	42 (13.1)	
≥37 months	458 (86.9)	69 (13.1)		96 (86.5)	15 (13.5)		554 (86.8)	84 (13.2)	
Major^b^, *n* (%)			0.891			0.003			0.367
Art	378 (88.3)	50 (11.7)		34 (75.6)	11 (24.4)		412 (87.1)	61 (12.9)	
Science	863 (88.1)	117 (11.9)		256 (90.8)	26 (9.2)		1119 (88.7)	143 (11.3)	
Year of study^b^, *n* (%)			0.430			0.282			0.364
Year one	387 (89.6)	45 (10.4)		104 (91.2)	10 (8.8)		491 (89.9)	55 (10.1)	
Year two	339 (88.7)	43 (11.3)		109 (87.9)	15 (12.1)		448 (88.5)	58 (11.5)	
Year three	404 (87.4)	58 (12.6)		63 (84.0)	12 (16.0)		467 (87.0)	70 (13.0)	
Year four	110 (84.6)	20 (15.4)		11 (100.0)	0 (0.0)		121 (85.8)	20 (14.2)	
Grade point average^b^, *n* (%)			0.074			0.164			0.043
< 2.50	75 (85.2)	13 (14.8)		51 (87.9)	7 (12.1)		126 (86.3)	20 (13.7)	
2.50–2.99	256 (91.8)	23 (8.2)		105 (90.5)	11 (9.5)		361 (91.4)	34 (8.6)	
3.00–3.49	461 (86.8)	70 (13.2)		81 (83.5)	16 (16.5)		542 (86.3)	86 (13.7)	
3.50–3.99	365 (87.3)	53 (12.7)		40 (97.6)	1 (2.4)		405 (88.2)	54 (11.8)	
≥ 4.00	82 (94.3)	5 (5.7)		12 (92.3)	1 (7.7)		94 (94.0)	6 (6.0)	
Social withdrawal^b^, *n* (%)			<0.001			0.020			<0.001
Yes	22 (66.7)	11 (33.3)		17 (73.9)	6 (26.1)		39 (69.6)	17 (30.4)	
No	1219 (88.7)	156 (11.3)		273 (89.8)	31 (10.2)		1492 (88.9)	187 (11.1)	
Hope^a^, mean (s.d.)	4.80 (1.24)	3.92 (1.42)	<0.001	4.92 (1.25)	3.86 (1.18)	<0.001	4.82 (1.24)	3.91 (1.38)	<0.001
Flourishing^a^, mean (s.d.)	40.62 (7.86)	33.77 (8.47)	<0.001	40.76 (8.04)	31.46 (6.26)	<0.001	40.64 (7.89)	33.35 (8.15)	<0.001
Purpose in life^a^, mean (s.d.)	19.38 (4.47)	15.64 (5.23)	<0.001	19.49 (4.69)	14.14 (4.18)	<0.001	19.40 (4.51)	15.37 (5.08)	<0.001
Depression^a^, mean (s.d.)	6.13 (6.10)	15.20 (9.79)	<0.001	6.90 (7.39)	16.43 (8.58)	<0.001	6.28 (6.37)	15.42 (9.58)	<0.001
Anxiety^a^, mean (s.d.)	8.58 (6.58)	15.92 (9.11)	<0.001	8.56 (7.49)	13.78 (8.43)	<0.001	8.58 (6.76)	15.53 (9.02)	<0.001
Stress^a^, mean (s.d.)	10.91 (7.29)	17.89 (8.63)	<0.001	11.02 (8.03)	18.27 (7.85)	<0.001	10.93 (7.43)	17.96 (8.48)	<0.001
Mindset^a^, mean (s.d.)	2.82 (0.87)	3.69 (1.07)	<0.001	2.79 (0.93)	3.53 (0.82)	<0.001	2.81 (0.88)	3.66 (1.03)	<0.001
Seeking help^a^, mean (s.d.)	3.35 (0.46)	3.21 (0.50)	<0.001	3.22 (0.51)	3.05 (0.57)	0.064	3.33 (0.47)	3.18 (0.52)	<0.001

a. *t*-test indicated a significant difference between groups of no suicidal behaviour (no) and with suicidal behaviour (yes) if *P* < 0.05.

b. *χ*^2^-test indicated a significant difference between groups of no suicidal behaviour (no) and with suicidal behaviour (yes) if *P* < 0.05.

The overall prevalence rate of social withdrawal behaviours was 3.2% (7.0% for men and 2.3% for women, *P* < 0.001). The prevalence rate of prolonged social withdrawal behaviour among participants with a GPA < 2.5 was higher than other GPA groups. More year 3 male participants reported social withdrawal behaviours (16.0%). Participants who had social withdrawal behaviours had lower reported scores for hope, flourishing, attitude toward seeking professional help and purpose in life; more depression, anxiety and stress symptoms; and more fixed mindset of negative emotional states (all with *P* < 0.05) (see Table 4).

**Table 4:** Descriptive statistics of social withdrawal, by gender

	Female			Male			Total		
	No	Yes	*P*-value	No	Yes	*P*-value	No	Yes	*P*-value
Total^a^, *n* (%)	1375 (97.7)	33 (2.3)		304 (93.0)	23 (7.0)		1679 (96.8)	56 (3.2)	
Type of hometown^b^, *n* (%)			0.102			0.484			0.097
City	400 (99.0)	4 (1.0)		89 (94.7)	5 (5.3)		489 (98.2)	9 (1.8)	
Town	456 (97.0)	14 (3.0)		107 (93.9)	7 (6.1)		563 (96.4)	21 (3.6)	
Village	519 (97.2)	15 (2.8)		108 (90.8)	11 (9.2)		627 (96.0)	26 (4.0)	
Time of stay^b^, *n* (%)			0.211			0.291			0.274
≤12 months	327 (96.2)	13 (3.8)		78 (94.0)	5 (6.0)		405 (95.7)	18 (4.3)	
13–24 months	261 (97.8)	6 (2.2)		79 (91.9)	7 (8.1)		340 (96.3)	13 (3.7)	
25–36 months	269 (98.2)	5 (1.8)		41 (91.9)	6 (12.8)		310 (96.6)	11 (3.4)	
≥37 months	518 (98.3)	9 (1.7)		106 (95.5)	5 (4.5)		624 (97.8)	14 (2.2)	
Major^b^, *n* (%)			0.711			0.600			0.935
Art	417 (97.4)	11 (2.6)		41 (91.1)	4 (8.9)		458 (96.8)	15 (3.2)	
Science	958 (97.8)	22 (2.2)		263 (93.3)	19 (6.7)		1221 (96.8)	41 (3.2)	
Year of study^b^, *n* (%)			0.308			0.004			0.946
Year one	419 (97.0)	13 (3.0)		111 (97.4)	3 (2.6)		530 (97.1)	16 (2.9)	
Year two	374 (97.9)	8 (2.1)		116 (93.5)	8 (6.5)		490 (96.8)	16 (3.2)	
Year three	455 (98.5)	7 (1.5)		63 (84.0)	12 (16.0)		518 (96.5)	19 (3.5)	
Year four	125 (97.7)	5 (3.8)		11 (100.0)	0 (0.0)		136 (96.5)	5 (3.5)	
Grade point average^b^, *n* (%)			0.043			0.187			0.005
< 2.50	83 (94.3)	5 (5.7)		51 (87.9)	7 (12.1)		134 (91.8)	12 (8.2)	
2.50–2.99	268 (96.1)	11 (3.9)		113 (97.4)	3 (2.6)		381 (96.5)	14 (3.5)	
3.00–3.49	522 (98.3)	9 (1.7)		89 (91.8)	8 (8.2)		611 (97.3)	17 (2.7)	
3.50–3.99	412 (98.6)	6 (1.4)		38 (92.7)	3 (7.3)		450 (98.0)	9 (2.0)	
≥ 4.00	85 (97.7)	2 (2.3)		12 (92.3)	1 (7.7)		97 (97)	3 (3.0)	
Hope^a^, mean (s.d.)	4.72 (1.27)	3.56 (1.48)	<0.001	4.85 (1.27)	4.02 (1.33)	0.003	4.75 (1.28)	3.75 (1.43)	<0.001
Flourishing^a^, mean (s.d.)	40.02 (8.05)	30.76 (10.44)	<0.001	40.23 (8.11)	32.78 (9.09)	<0.001	40.06 (8.06)	31.59 (9.87)	<0.001
Purpose in life^a^, mean (s.d.)	19.07 (4.63)	13.42 (5.37)	<0.001	19.19 (4.79)	14.83 (5.01)	<0.001	19.09 (4.66)	14.00 (5.23)	<0.001
Depression^a^, mean (s.d.)	6.95 (6.92)	18.12 (11.60)	<0.001	7.18 (7.40)	18.43 (9.74)	<0.001	6.99 (7.01)	18.25 (10.78)	<0.001
Anxiety^a^, mean (s.d.)	9.24 (7.05)	18.48 (11.55)	<0.001	8.53 (7.34)	17.39 (8.62)	<0.001	9.11 (7.11)	18.04 (10.38)	<0.001
Stress^a^, mean (s.d.)	11.59 (7.69)	17.88 (9.50)	0.001	11.16 (7.85)	20.87 (9.28)	<0.001	11.52 (7.72)	19.11 (9.44)	<0.001
Mindset^a^, mean (s.d.)	2.90 (0.93)	3.81 (0.91)	<0.001	2.82 (0.94)	3.59 (0.73)	<0.001	2.89 (0.93)	3.72 (0.84)	<0.001
Seeking help^a^, mean (s.d.)	3.34 (0.46)	3.04 (0.48)	<0.001	3.22 (0.52)	2.98 (0.29)	0.001	3.32 (0.48)	3.01 (0.41)	<0.001

a. *t*-test indicated a significant difference between no social withdrawal (no) and social withdrawal (yes) if *P* < 0.05.

b. *χ*^2^-test indicated a significant difference between no social withdrawal (no) and social withdrawal (yes) if *P* < 0.05.

The prevalence rates of self-harm and suicidal behaviours among participants with social withdrawal behaviours were 26.8% and 30.4%, respectively. In general, participants who had engaged in self-harm and suicidal behaviours had lower reported scores for hope, flourishing, attitude toward seeking professional help and purpose in life; more depression, anxiety and stress symptoms; and more fixed mindset of negative emotional states (all with *P* < 0.05).

The associations between social withdrawal status and self-harm and suicidal behaviour are outlined in Tables 5 and 6, respectively. In general, before adjusting for psychological variables, social withdrawal status was significantly associated with self-harm (odds ratio 2.0, 95% CI 1.2–3.3) and suicidal behaviour (odds ratio 2.4, 95% CI 1.5–3.8). However, the associations were adjusted and became statistically insignificant after adjustment for psychological factors in the final models. In addition, flourishing (odds ratio 0.96, 95% CI 0.93–0.99) and stress (odds ratio 1.04, 95% CI 1.01–1.08) were the two remaining significant variables with self-harm in the final models; flourishing (odds ratio 0.96, 95% CI 0.92–0.99), depression (odds ratio 1.09, 95% CI 1.05–1.21) and fixed mindset of negative emotional states (odds ratio 1.38, 95% CI 1.07–1.77) were the three remaining significant variables with suicidal behaviour in the final models.

**Table 5:** Logistic regression on self-harm among male and female participants

	Female (*n* = 1408)		Male (*n* = 327)		Total (*n* = 1735)	
	Odds ratio (95% CI)	*P*-value	Odds ratio (95% CI)	*P*-value	Odds ratio (95% CI)	*P*-value
Crude model						
Social withdrawal	1.27 (0.64–2.53)	0.496	3.02 (1.52–6.74)	0.002	1.97 (1.22–3.19)	0.006
Model 1^a^						
Social withdrawal	1.34 (0.67–2.72)	0.403	3.18 (1.38–7.34)	0.007	2.00 (1.22–3.29)	0.006
Model 2^b^						
Social withdrawal	0.46 (0.21–1.03)	0.059	1.38 (0.52–3.62)	0.522	0.84 (0.48–1.48)	0.553
Hope	1.20 (0.95–1.51)	0.130	0.93 (0.58–1.49)	0.746	1.12 (0.92–1.37)	0.253
Flourishing	0.95 (0.91–0.99)	0.007	0.98 (0.90–1.07)	0.710	0.96 (0.93–0.99)	0.021
Purpose in life	0.99 (0.93–1.06)	0.813	0.98 (0.85–1.12)	0.749	0.99 (0.93–1.05)	0.666
Depression	1.03 (1.00–1.07)	0.094	0.98 (0.92–1.06)	0.629	1.02 (0.99–1.06)	0.141
Anxiety	1.03 (0.99–1.07)	0.102	1.03 (0.96–1.11)	0.411	1.02 (0.99–1.06)	0.134
Stress	1.04 (1.00–1.07)	0.062	1.08 (1.00–1.17)	0.064	1.04 (1.01–1.08)	0.009
Mindset	1.17 (0.89–1.53)	0.271	0.83 (0.50–1.39)	0.478	1.04 (0.83–1.32)	0.718
Seeking help	0.92 (0.61–1.40)	0.705	0.72 (0.34–1.51)	0.384	0.80 (0.57–1.13)	0.211

a. Model 1 was adjusted for the demographic characteristics, including type of hometown, time of stay in the current city, major, year of study and grade point average.

b. Model 2 was adjusted for the factors in model 1 and then adjusted for hope, flourishing, purpose in life, depression, anxiety, stress, mindset and seeking help.

**Table 6:** Logistic regression on suicidal behaviour among male and female participants

	Female (*n* = 1408)		Male (*n* = 327)		Total (*n* = 1735)	
	Odds ratio (95% CI)	*P*-value	Odds ratio (95% CI)	*P*-value	Odds ratio (95% CI)	*P*-value
Crude model						
Social withdrawal	2.09 (1.17–3.71)	0.013	2.99 (1.32–6.73)	0.008	2.29 (1.44–3.63)	<0.001
Model 1^a^						
Social withdrawal	2.35 (1.30–4.27)	0.005	2.22 (0.89–5.52)	0.087	2.35 (1.45–3.81)	0.001
Model 2^b^						
Social withdrawal	0.63 (0.30–1.34)	0.229	0.45 (0.12–1.65)	0.228	0.74 (0.41–1.34)	0.318
Hope	1.22 (0.96–1.54)	0.106	1.55 (0.88–2.74)	0.130	1.22 (0.98–1.50)	0.070
Flourishing	0.96 (0.92–1.00)	0.045	0.91 (0.83–1.00)	0.051	0.96 (0.92–0.99)	0.011
Purpose in life	0.97 (0.90–1.03)	0.305	0.88 (0.75–1.02)	0.092	0.96 (0.90–1.02)	0.148
Depression	1.09 (1.05–1.13)	<0.001	1.06 (0.98–1.15)	0.169	1.09 (1.05–1.21)	<0.001
Anxiety	1.03 (0.99–1.07)	0.101	0.93 (0.86–1.01)	0.124	1.01 (0.98–1.05)	0.454
Stress	0.99 (0.95–1.03)	0.635	1.11 (1.01–1.23)	0.039	1.00 (0.97–1.04)	0.893
Mindset	1.46 (1.10–1.93)	0.008	1.01 (0.53–1.95)	0.968	1.38 (1.07–1.77)	0.013
Seeking help	0.97 (0.64–1.47)	0.885	0.65 (0.28–1.52)	0.318	0.92 (0.64–1.32)	0.645

a. Model 1 was adjusted for demographic characteristics, including type of hometown, time of stay in the current city, major, year of study and grade point average.

b. Model 2 was additionally adjusted for hope, flourishing, purpose in life, depression, anxiety, stress, mindset and seeking help.

The associations between suicidal behaviour and social withdrawal status among women and men were both significant in the crude models (women: odds ratio 2.09, 95% CI 1.17–3.71; men: odds ratio 2.99, 95% CI 1.32–6.73), but became insignificant in the final models for both genders. Among women, social withdrawal status was not associated with self-harm regardless of the level of adjustment (all *P* > 0.05). Furthermore, among female participants, type of major (science: odds ratio 1.85, 95% CI 1.19–2.80), flourishing (odds ratio 0.96, 95% CI 0.96–0.99), depression (odds ratio 1.09, 95% CI 1.07–1.13) and mindset of negative emotional states (odds ratio 1.46, 95% CI 1.10–1.93) were the remaining significant variables with suicidal behaviour. Among men, social withdrawal status (odds ratio 3.18, 95% CI 1.38–7.34) was associated with self-harm, but was attenuated after being adjusted for psychological variables (odds ratio 1.38, 95% CI 0.52–3.62) (see Table 5). In addition, type of major (science: odds ratio 0.19, 95% CI 0.06–0.60) and stress (odds ratio 1.11, 95% CI 1.01–1.23) among male participants were the remaining significant variables with suicidal behaviour (see Table 6) in the final models (see Supplementary Tables 2a–c and 3a–c available at https://doi.org/10.1192/bjo.2021.47, for a detailed breakdown by gender).

## Discussion

A cross-sectional survey, with a relatively rigorous recruitment method compared with convenient sampling, was conducted to examine the prevalence of self-harm, suicidal behaviour and prolonged withdrawal behaviour among Chinese university students. We also investigated the potential additional risk of social withdrawal behaviour among those with self-harm or suicidal behaviour related to poor mental health status among female and male university students. The data reveals that 11.8% of our study participants engaged in either self-harm or suicidal behaviour in the past year. Only a small percentage (3.2%) of the sample reported having prolonged social withdrawal behaviour for longer than 3 months, and self-harm and suicidal behaviour were prevalent among the withdrawn students. In general, a recent positive outlook for the future (i.e. flourishing) and a negative attitude about a change in poor psychological well-being (i.e. depression and stress) have stronger associations or predictive values with self-harm and suicidal behaviour than withdrawal behaviour. Gender differences, year level of study and type of major have different effects on men and women who report self-harm and suicidal behaviour.

### Self-harm and suicidal behaviour

The prevalence rates of self-harm and suicidal behaviour found in our study are comparable with studies conducted across the globe^36^ and within the Chinese student community.^37^ Previous studies have suggested that university students in China are more prone to exhibiting self-harm and suicidal behaviour because of the extremely competitive education system and labour market in contemporary China. Furthermore, university students are emerging adults who face the challenges of leaving home and building independence and self-discipline. Students who have difficulties in adapting to university life (year 1), anticipate the challenges of transiting to the job market (year 3), and those with a lower GPA, may have higher stress and anxiety and exhibit more social withdrawal, self-harm and suicidal behaviours. Accordingly, our study found supporting evidence to suggest that self-harm and suicidal behaviour in Chinese university students require greater attention, especially as there were approximately 28.3 million university students in 2018.^15,38^

### Social withdrawal behaviour and suicidal behaviour

Prolonged social withdrawal behaviour among young people is a relatively new research area, and only a handful of studies have been conducted in China.^39^ In addition, only a few studies focused on social withdrawal behaviour have reported quantitative findings to suggest an association with self-harm and suicidal behaviour. Wong et al^11^ were possibly the first research group to find that people aged 12–29 years who engage in withdrawal behaviour for longer than 6 months are around 2.3 times more likely to engage in intentional self-injury behaviour. Yong and Nomura^12^ reported that individuals aged 15–39 years who engage in prolonged social withdrawal behaviour were 1.2 times more likely to have suicide risks. Thus far, apart from our study, only one study has reported the gender difference in the association between self-harm and suicidal behaviour and prolonged social withdrawal behaviour. Yong et al^40^ conducted a study of people aged 15–64 years living in rural areas in Japan, and found that men with prolonged social withdrawal behaviour were more likely to report poor physical condition, feelings of distress and passive suicidal ideation than men with no withdrawal behaviour. However, this is not the case for withdrawn women. In other words, it seems that our data also support our hypothesis that there is an association between self-harm and social withdrawal behaviour, and there is also gender difference in the associations. It was suggested that the traditional Japanese culture and expectations for men and women, and the availability of social support, are some of the factors that contribute to the gender differences of people with social withdrawal behaviour with suicide risks. In the final models of our study, it seems that factors like academic performance, years of study at university and type of major have different effects on the association of self-harm and withdrawal behaviour among male and female university students. However, regardless of gender, a more positive future outlook seems to protect university student participants from engaging in such risky behaviours, and a negative perception toward changes in poor psychological well-being perpetuates their engagement in such risky behaviours.

### Possible explanations for the association

It is well-understood that youth self-harm and suicidal behaviour are multifaceted public health issues that involve individual factors (e.g. restricted academic achievement, perfectionism, low optimism personality characteristics), interpersonal factors (e.g. bullying, poor peer and familial relationships) and societal factors (e.g. lack of employment opportunities, stigmatisation).^13^ Li and Wong^14^ also found that prolonged social withdrawal behaviour is associated with similar risk factors for suicidal behaviour. Hence, it is conceivable that part of the association between self-harm and social withdrawal behaviour stems from a shared association with other identified risk factors. Young people who engage in self-harm and withdrawal behaviour may have more psychological distress, and use these behaviours as their coping mechanisms.

Furthermore, the association between social withdrawal status and self-harm/suicide could be confounded by mental health status. When adjusted for DASS-21 score, the association between social withdrawal behaviour and self-harm or suicidal behaviour becomes insignificant. One explanation is that poor mental health status causes both social withdrawal and self-harm and suicidal behaviour. From this perspective, it may be that social withdrawal is indirectly associated with self-harm and suicidal behaviour, and both youth risk behaviours potentially have a similar function that allows them to remain in a comfort zone and disconnect from the perceived harsh living conditions, at least in the short term. However, in the longer term, the psychological pain or ‘psych-ache’ of people with chronic suicidal beliefs^41^ and the inability to leave the secluded environment of the withdrawal^14^ may exacerbate the impairments, although more inquiry is needed before conclusions can be made. In other words, the withdrawn students who participated in the study had high levels of depression, anxiety and stress, which could be the predominant association with suicidal behaviour, independent of their withdrawn status.

### Limitations

First, the definition of prolonged social withdrawal behaviour used in this study may be different from previous studies, because there are not yet consensual clinical and research definitions of *hikikomori* or prolonged social withdrawal behaviour.^8^ In terms of clinical research, a diagnostic interview or a self-reported screening questionnaire for psychiatric morbidity is recommended to assess the presence of psychiatric disorders. This is required to rule out the influence of severe psychiatric disorders in the development social withdrawal behaviour. However, regarding the purpose of this study, limitations in availability of funding and the feasibility of having trained clinicians to conduct clinical interviews with such a large sample, our definition seems to be the most appropriate definition of prolonged social withdrawal behaviour as cited in the scientific literature. Second, this study relied on self-reported data, but we attempted to include various measures to enhance the reliability and quality of the collected data. Third, some key factors about self-harm and withdrawal behaviour, such as a history of psychiatric issues and current psychopathology, were not included in the survey. Fourth, the small number of reported suicide attempts and specific self-harm behaviours were insufficient for further comparison between the two genders in specific types of behaviours. Given the limited number of available psychiatrists and psychologists in China^42^ to ascertain the psychopathological status comprehensively, it is difficult to assume that the inclusion of these key factors in the survey could provide accurate information that is representative of the situation in the studied area and among the studied population. Despite these limitations, this study is one of the first to explore the presence of, and associations between, self-harm and suicidal behaviour and prolonged social withdrawal behaviour, with a focus on gender difference, among a large sample of university students in China.

In conclusion, the current study found that almost 12% of the university students in a province in China engaged in either self-harm or suicidal behaviours. It is of concern that over 25% of those who have prolonged social withdrawal behaviour engage in self-harm or suicidal behaviour. Given the difficulty in identifying and intervening in self-harm behaviour with young people, there are preventive and clinical implications in the knowledge that social withdrawal behaviour is a high-risk factor for self-harm. University teachers and administrative colleagues with heightened awareness of university students who have missed teaching and learning activities for a period of time may provide an opportunity for identification and subsequent intervention for self-harm and suicidal behaviour.

The findings from this study may also help educators, social workers and clinicians who work with young people engaged in school drop-out or truancy, to ask about the level of suicide risk. The findings also highlight the need for additional research in the shared risk factors and causal association between self-harm and social withdrawal behaviour among different genders in different contexts, using longitudinal, quantitative and in-depth qualitative research methodologies.

The potential of health and societal costs introduced to help withdrawn and suicidal individuals with their re-integration into society are substantial. However, if this phenomenon remains unrecognised and understudied by public and research communities at its early stage of emergence, many countries may experience a significant loss of the skills and resources that these young people could have contributed to the world in general.

## Data Availability

The data that support the findings of this study are available from the corresponding author, P.W.C.W., upon reasonable request.

## Supplementary material

For supplementary material accompanying this paper visit http://dx.doi.org/10.1192/bjo.2021.47. click here to view supplementary material
