# Supplementary material for: Investigating prolonged social withdrawal behaviour as a risk factor for self-harm and suicidal behaviours
Source: BJPsych Open. 2021 Apr 30;7(3):e90. doi: 10.1192/bjo.2021.47 (PMC8142544; doi:10.1192/bjo.2021.47)
Supplement: Supplementary file 1 [file S2056472421000478sup001.docx]

**Supplementary Table 1** Frequency and percentage of social withdrawal measure in details by gender

|  | Spend all day at home | | |  | Avoid social occasions and access to people | | |
| --- | --- | --- | --- | --- | --- | --- | --- |
|  | Female  (n = 1408) | Male  (n = 327) | Total  (n = 1735) |  | Female  (n = 1408) | Male  (n = 327) | Total  (n = 1735) |
| Yes, n (%) | 444 (31.5) | 123 (37.6) | 567 (32.7) |  | 271 (19.2) | 70 (21.4) | 341 (19.7) |
| Duration, n (%) |  |  |  |  |  |  |  |
| ≤ 3 months | 191 (13.6) | 41 (12.5) | 232 (13.4) |  | 84 (6.0) | 17 (5.2) | 101 (5.8) |
| 3-6 months | 64 (4.5) | 17 (5.2) | 81 (4.7) |  | 32 (2.3) | 10 (3.1) | 42 (2.4) |
| 6-12 months | 37 (2.6) | 13 (4.0) | 50 (2.9) |  | 22 (1.6) | 8 (2.4) | 30 (1.7) |
| ≥ 12 months | 152 (10.8) | 52 (15.9) | 204 (11.8) |  | 133 (9.4) | 35 (10.7) | 168 (9.7) |
| Feel troubled, n (%) | 145 (10.3) | 60 (18.3) | 205 (11.8) |  | 161 (11.4) | 46 (14.1) | 207 (11.9) |
| Cause difficulty in daily life^b^, mean (s.d) | 1.68 (0.67) | 1.98 (0.89) | 1.75 (0.74) |  | 1.98 (0.69) | 2.19 (0.86) | 2.02 (0.73) |
| Influence on life^c^, n (%) |  |  |  |  |  |  |  |
| Academic | 218 (15.5) | 78 (23.9) | 296 (17.1) |  | 147 (10.4) | 31 (9.5) | 178(10.3) |
| Work | 156 (11.1) | 52 (15.9) | 208 (12.0) |  | 102 (7.2) | 25 (7.6) | 127 (7.3) |
| Financial situation | 173 (12.3) | 57 (17.4) | 230 (13.3) |  | 65 (4.6) | 18 (5.5) | 83 (4.8) |
| Social life | 267 (19.0) | 67 (20.5) | 334 (19.3) |  | 217 (15.4) | 57 (17.4) | 274 (15.8) |
| Health | 170 (12.1) | 44 (13.5) | 214 (12.3) |  | 62 (4.4) | 23 (7.0) | 85 (4.9) |
| Family relationship | 81 (5.8) | 24 (7.3) | 105 (6.1) |  | 61 (4.3) | 16 (4.9) | 77 (4.4) |
| Boyfriend/girlfriend relationship | 56 (4.0) | 37 (11.3) | 93 (5.4) |  | 41 (2.9) | 17 (5.2) | 58 (3.3) |
| Psychological development | 187 (13.3) | 45 (13.8) | 232 (13.4) |  | 159 (11.3) | 39 (11.9) | 198 (11.4) |

a. Feel troubled was measured by a dichotomous Yes/No question “*Do you feel disturb by the situation?*”

b. Cause difficulty in daily was measured by the question “Will it make any trouble for your work, study, daily housework or interpersonal relationship?” applying a 4-point Likert scale: 1 = *No difficulty at all*, 4 = *Extremely difficult*.

c. Influence on life was measured by the multiple answers question “*What kind of influence has this situation affected on your life?*”

**Supplementary Table 2a** Logistic regression on self-harm among female participants

|  | Female (n=1408) | | | | | | | |
| --- | --- | --- | --- | --- | --- | --- | --- | --- |
|  | Crude model | |  | Model 1 | |  | Model 2 | |
|  | OR (95% CI) | *P-value* |  | OR (95% CI) | *P-value* |  | OR (95% CI) | *P-value* |
| Social withdrawal |  |  |  |  |  |  |  |  |
| Yes | 1.27 (0.64, 2.53) | 0.496 |  | 1.34 (0.67, 2.72) | 0.403 |  | 0.46 (0.21, 1.03) | 0.059 |
| No | Reference |  |  | Reference |  |  | Reference |  |
| Type of hometown |  |  |  |  |  |  |  |  |
| City | - | - |  | Reference |  |  | Reference |  |
| Town | - | - |  | 0.90 (0.59, 1.36) | 0.601 |  | 0.95 (0.61, 1.48) | 0.818 |
| Village | - | - |  | 0.58 (0.37, 0.91) | 0.108 |  | 0.56 (0.34, 0.90) | 0.018 |
| Time of stay |  |  |  |  |  |  |  |  |
| ≤ 12 months | - | - |  | Reference |  |  | Reference |  |
| 13-24 months | - | - |  | 1.04 (0.50, 2.18) | 0.917 |  | 1.31 (0.59, 2.90) | 0.514 |
| 25-36 months | - | - |  | 1.09 (0.53, 2.24) | 0.813 |  | 1.36 (0.63, 2.93) | 0.439 |
| ≥ 37 months | - | - |  | 0.88 (0.51, 1.52) | 0.651 |  | 1.04 (0.59, 1.85) | 0.890 |
| Major |  |  |  |  |  |  |  |  |
| Art | - | - |  | Reference |  |  | Reference |  |
| Science | - | - |  | 0.88 (0.61, 1.29) | 0.512 |  | 1.11 (0.74, 1.66) | 0.615 |
| Year of study |  |  |  |  |  |  |  |  |
| Year one | - | - |  | Reference |  |  | Reference |  |
| Year two | - | - |  | 0.70 (0.37, 1.35) | 0.287 |  | 0.48 (0.24, 0.97) | 0.040 |
| Year three | - | - |  | 0.72 (0.39, 1.33) | 0.293 |  | 0.48 (0.25, 0.94) | 0.033 |
| Year four | - | - |  | 1.25 (0.62, 2.52) | 0.528 |  | 1.13 (0.54, 2.36) | 0.757 |
| Grade Point Average |  |  |  |  |  |  |  |  |
| GPA < 2.50 | - | - |  | Reference |  |  | Reference |  |
| GPA 2.50-2.99 | - | - |  | 0.95 (0.44, 2.05) | 0.886 |  | 1.16 (0.50, 2.68) | 0.733 |
| GPA 3.00-3.49 | - | - |  | 1.13 (0.53, 2.39) | 0.751 |  | 1.47 (0.65, 3.32) | 0.353 |
| GPA 3.50-3.99 | - | - |  | 1.06 (0.49, 2.33) | 0.877 |  | 1.76 (0.75, 4.12) | 0.192 |
| GPA ≥ 4.00 | - | - |  | 0.57 (0.12, 1.73) | 0.319 |  | 0.88 (0.27, 2.83) | 0.830 |
| Hope | - | - |  | - | - |  | 1.20 (0.95, 1.51) | 0.130 |
| Flourishing | - | - |  | - | - |  | 0.95 (0.91, 0.99) | 0.007 |
| Purpose in life | - | - |  | - | - |  | 0.99 (0.93, 1.06) | 0.813 |
| Depression | - | - |  | - | - |  | 1.03 (1.00, 1.07) | 0.094 |
| Anxiety | - | - |  | - | - |  | 1.03 (0.99, 1.07) | 0.102 |
| Stress | - | - |  | - | - |  | 1.04 (1.00, 1.07) | 0.062 |
| Mindsets | - | - |  | - | - |  | 1.17 (0.89, 1.53) | 0.271 |
| Seeking help | - | - |  | - | - |  | 0.92 (0.61, 1.40) | 0.705 |

**Supplementary Table 2b** Logistic regression on self-harm among male participants

|  | Male (n=327) | | | | | | | |
| --- | --- | --- | --- | --- | --- | --- | --- | --- |
|  | Crude model | |  | Model 1 | |  | Model 2 | |
|  | OR (95% CI) | *P-value* |  | OR (95% CI) | *P-value* |  | OR (95% CI) | *P-value* |
| Social withdrawal |  |  |  |  |  |  |  |  |
| Yes | 3.02 (1.52, 6.74) | 0.002 |  | 3.18 (1.38, 7.34) | 0.007 |  | 1.38 (0.52, 3.62) | 0.522 |
| No | Reference |  |  | Reference |  |  | Reference |  |
| Type of hometown |  |  |  |  |  |  |  |  |
| City | - | - |  | Reference |  |  | Reference |  |
| Town | - | - |  | 0.61 (0.25, 1.52) | 0.289 |  | 0.59 (0.22, 1.61) | 0.303 |
| Village | - | - |  | 1.06 (0.45, 2.49) | 0.892 |  | 1.10 (0.42, 2.88) | 0.847 |
| Time of stay |  |  |  |  |  |  |  |  |
| ≤ 12 months | - | - |  | Reference |  |  | Reference |  |
| 13-24 months | - | - |  | 0.93 (0.22, 3.85) | 0.915 |  | 0.86 (0.20, 3.79) | 0.843 |
| 25-36 months | - | - |  | 0.36 (0.08, 1.59) | 0.178 |  | 0.28 (0.06, 1.32) | 0.107 |
| ≥ 37 months | - | - |  | 0.48 (0.14, 1.61) | 0.234 |  | 0.43 (0.12, 1.59) | 0.209 |
| Major |  |  |  |  |  |  |  |  |
| Art | - | - |  | Reference |  |  | Reference |  |
| Science | - | - |  | 0.39 (0.16, 0.92) | 0.032 |  | 0.31 (0.12, 0.82) | 0.017 |
| Year of study |  |  |  |  |  |  |  |  |
| Year one | - | - |  | Reference |  |  | Reference |  |
| Year two | - | - |  | 2.16 (0.56, 8.25) | 0.261 |  | 1.33 (0.33, 5.44) | 0.688 |
| Year three | - | - |  | 5.53 (1.52, 20.12) | 0.009 |  | 5.48 (1.38, 21.69) | 0.015 |
| Year four | - | - |  | 11.17 (1.94, 64.37) | 0.007 |  | 12.26 (1.81, 83.29) | 0.010 |
| Grade Point Average |  |  |  |  |  |  |  |  |
| GPA < 2.50 | - | - |  | Reference |  |  | Reference |  |
| GPA 2.50-2.99 | - | - |  | 0.60 (0.24, 1.49) | 0.267 |  | 0.46 (0.17, 1.25) | 0.125 |
| GPA 3.00-3.49 | - | - |  | 0.49 (0.19, 1.27) | 0.141 |  | 0.51 (0.19, 1.39) | 0.189 |
| GPA 3.50-3.99 | - | - |  | 2.59 (0.07, 1.00) | 0.050 |  | 0.24 (0.05, 1.09) | 0.065 |
| GPA ≥ 4.00 | - | - |  | N/A |  |  | N/A |  |
| Hope | - | - |  | - | - |  | 0.93 (0.58, 1.49) | 0.746 |
| Flourishing | - | - |  | - | - |  | 0.98 (0.90, 1.07) | 0.710 |
| Purpose in life | - | - |  | - | - |  | 0.98 (0.85, 1.12) | 0.749 |
| Depression | - | - |  | - | - |  | 0.98 (0.92, 1.06) | 0.629 |
| Anxiety | - | - |  | - | - |  | 1.03 (0.96, 1.11) | 0.411 |
| Stress | - | - |  | - | - |  | 1.08 (1.00, 1.17) | 0.064 |
| Mindsets | - | - |  | - | - |  | 0.83 (0.50, 1.39) | 0.478 |
| Seeking help | - | - |  | - | - |  | 0.72 (0.34, 1.51) | 0.384 |

Su**pplementary Table 2c** Logistic regression on self-harm among all participants

|  | Total (n=1,735) | | | | | | | |
| --- | --- | --- | --- | --- | --- | --- | --- | --- |
|  | Crude model | |  | Model 1 | |  | Model 2 | |
|  | OR (95% CI) | *P-value* |  | OR (95% CI) | *P-value* |  | OR (95% CI) | *P-value* |
| Social withdrawal |  |  |  |  |  |  |  |  |
| Yes | 1.97 (1.22, 3.19) | 0.006 |  | 2.00 (1.22, 3.29) | 0.006 |  | 0.84 (0.48, 1.48) | 0.553 |
| No | Reference |  |  | Reference |  |  | Reference |  |
| Type of hometown |  |  |  |  |  |  |  |  |
| City | - | - |  | Reference |  |  | Reference |  |
| Town | - | - |  | 0.81 (0.56, 1.18) | 0.279 |  | 0.80 (0.54, 1.19) | 0.269 |
| Village | - | - |  | 0.66 (0.44, 0.97) | 0.034 |  | 0.61 (0.40, 0.92) | 0.019 |
| Time of stay |  |  |  |  |  |  |  |  |
| ≤ 12 months | - | - |  | Reference |  |  | Reference |  |
| 13-24 months | - | - |  | 1.07 (0.57, 2.02) | 0.838 |  | 1.18 (0.60, 2.31) | 0.631 |
| 25-36 months | - | - |  | 0.85 (0.45, 1.61) | 0.626 |  | 0.93 (0.47, 1.81) | 0.820 |
| ≥ 37 months | - | - |  | 0.79 (0.48, 1.28) | 0.338 |  | 0.86 (0.51, 1.43) | 0.560 |
| Major |  |  |  |  |  |  |  |  |
| Art | - | - |  | Reference |  |  | Reference |  |
| Science | - | - |  | 0.79 (0.56, 1.11) | 0.178 |  | 0.93 (0.65, 1.34) | 0.695 |
| Year of study |  |  |  |  |  |  |  |  |
| Year one | - | - |  | Reference |  |  | Reference |  |
| Year two | - | - |  | 0.93 (0.52, 1.64) | 0.793 |  | 0.67 (0.37, 1.21) | 0.182 |
| Year three | - | - |  | 1.21 (0.70, 2.09) | 0.497 |  | 0.94 (0.52, 1.68) | 0.832 |
| Year four | - | - |  | 1.84 (0.96, 3.51) | 0.065 |  | 1.69 (0.86, 3.34) | 0.129 |
| Grade Point Average |  |  |  |  |  |  |  |  |
| GPA < 2.50 | - | - |  | Reference |  |  | Reference |  |
| GPA 2.50-2.99 | - | - |  | 0.74 (0.42, 1.30) | 0.295 |  | 0.77 (0.43, 1.40) | 0.398 |
| GPA 3.00-3.49 | - | - |  | 0.74 (0.43, 1.29) | 0.289 |  | 0.83 (0.46, 1.48) | 0.523 |
| GPA 3.50-3.99 | - | - |  | 0.63 (0.35, 1.14) | 0.124 |  | 0.85 (0.45, 1.59) | 0.602 |
| GPA ≥ 4.00 | - | - |  | 0.31 (0.12, 0.83) | 0.020 |  | 0.42 (0.15, 1.15) | 0.090 |
| Hope | - | - |  | - | - |  | 1.12 (0.92, 1.37) | 0.253 |
| Flourishing | - | - |  | - | - |  | 0.96 (0.93, 0.99) | 0.021 |
| Purpose in life | - | - |  | - | - |  | 0.99 (0.93, 1.05) | 0.666 |
| Depression | - | - |  | - | - |  | 1.02 (0.99, 1.06) | 0.141 |
| Anxiety | - | - |  | - | - |  | 1.02 (0.99, 1.06) | 0.134 |
| Stress | - | - |  | - | - |  | 1.04 (1.01, 1.08) | 0.009 |
| Mindsets | - | - |  | - | - |  | 1.04 (0.83, 1.32) | 0.718 |
| Seeking help | - | - |  | - | - |  | 0.80 (0.57, 1.13) | 0.211 |

**Supplementary Table 3a** Logistic regression on suicidal behaviour among female participants

|  | Female (n=1,408) | | | | | | | |
| --- | --- | --- | --- | --- | --- | --- | --- | --- |
|  | Crude model | |  | Model 1 | |  | Model 2 | |
|  | OR (95% CI) | *P-value* |  | OR (95% CI) | *P-value* |  | OR (95% CI) | *P-value* |
| Social withdrawal |  |  |  |  |  |  |  |  |
| Yes | 2.09 (1.17, 3.71) | 0.013 |  | 2.35 (1.30, 4.27) | 0.005 |  | 0.63 (0.30, 1.34) | 0.229 |
| No | Reference |  |  | Reference |  |  | Reference |  |
| Type of hometown |  |  |  |  |  |  |  |  |
| City | - | - |  | Reference |  |  | Reference |  |
| Town | - | - |  | 0.70 (0.46, 1.06) | 0.094 |  | 0.69 (0.43, 1.11) | 0.125 |
| Village | - | - |  | 0.78 (0.51, 1.18) | 0.235 |  | 0.78 (0.49, 1.26) | 0.306 |
| Time of stay |  |  |  |  |  |  |  |  |
| ≤ 12 months | - | - |  | Reference |  |  | Reference |  |
| 13-24 months | - | - |  | 0.51 (0.25, 1.06) | 0.071 |  | 0.56 (0.24, 1.29) | 0.176 |
| 25-36 months | - | - |  | 1.16 (0.58, 2.31) | 0.675 |  | 1.53 (0.70, 3.34) | 0.286 |
| ≥ 37 months | - | - |  | 0.97 (0.56, 1.67) | 0.901 |  | 1.15 (0.62, 2.13) | 0.657 |
| Major |  |  |  |  |  |  |  |  |
| Art | - | - |  | Reference |  |  | Reference |  |
| Science | - | - |  | 1.22 (0.84, 1.79) | 0.303 |  | 1.85 (1.19, 2.86) | 0.006 |
| Year of study |  |  |  |  |  |  |  |  |
| Year one | - | - |  | Reference |  |  | Reference |  |
| Year two | - | - |  | 1.57 (0.85, 2.91) | 0.150 |  | 1.09 (0.54, 2.23) | 0.807 |
| Year three | - | - |  | 1.13 (0.61, 2.10) | 0.690 |  | 0.72 (0.36, 1.46) | 0.369 |
| Year four | - | - |  | 1.89 (0.94, 3.81) | 0.074 |  | 1.73 (0.78, 3.82) | 0.177 |
| Grade Point Average |  |  |  |  |  |  |  |  |
| GPA < 2.50 | - | - |  | Reference |  |  | Reference |  |
| GPA 2.50-2.99 | - | - |  | 0.54 (0.26, 1.14) | 0.104 |  | 0.65 (0.28, 1.52) | 0.319 |
| GPA 3.00-3.49 | - | - |  | 0.90 (0.46, 1.79) | 0.769 |  | 1.35 (0.61, 2.98) | 0.458 |
| GPA 3.50-3.99 | - | - |  | 0.84 (0.41, 1.71) | 0.624 |  | 1.71 (0.75, 3.93) | 0.203 |
| GPA ≥ 4.00 | - | - |  | 0.30 (0.10, 0.94) | 0.038 |  | 0.52 (0.15, 1.76) | 0.294 |
| Hope | - | - |  | - | - |  | 1.22 (0.96, 1.54) | 0.106 |
| Flourishing | - | - |  | - | - |  | 0.96 (0.92, 1.00) | 0.045 |
| Purpose in life | - | - |  | - | - |  | 0.97 (0.90, 1.03) | 0.305 |
| Depression | - | - |  | - | - |  | 1.09 (1.05, 1.13) | <0.001 |
| Anxiety | - | - |  | - | - |  | 1.03 (0.99, 1.07) | 0.101 |
| Stress | - | - |  | - | - |  | 0.99 (0.95, 1.03) | 0.635 |
| Mindsets | - | - |  | - | - |  | 1.46 (1.10, 1.93) | 0.008 |
| Seeking help | - | - |  | - | - |  | 0.97 (0.64, 1.47) | 0.885 |

**Supplementary Table 3b** Logistic regression on suicidal behaviour among male participants

|  | Male (n=327) | | | | | | | |
| --- | --- | --- | --- | --- | --- | --- | --- | --- |
|  | Crude model | |  | Model 1 | |  | Model 2 | |
|  | OR (95% CI) | *P-value* |  | OR (95% CI) | *P-value* |  | OR (95% CI) | *P-value* |
| Social withdrawal |  |  |  |  |  |  |  |  |
| Yes | 2.99 (1.32, 6.73) | 0.008 |  | 2.22 (0.89, 5.52) | 0.087 |  | 0.45 (0.12, 1.65) | 0.228 |
| No | Reference |  |  | Reference |  |  | Reference |  |
| Type of hometown |  |  |  |  |  |  |  |  |
| City | - | - |  | Reference |  |  | Reference |  |
| Town | - | - |  | 1.11 (0.41, 3.01) | 0.839 |  | 0.88 (0.26, 2.96) | 0.834 |
| Village | - | - |  | 1.62 (0.61, 4.31) | 0.334 |  | 1.39 (0.41, 4.68) | 0.600 |
| Time of stay |  |  |  |  |  |  |  |  |
| ≤ 12 months | - | - |  | Reference |  |  | Reference |  |
| 13-24 months | - | - |  | 0.93 (0.21, 4.12) | 0.920 |  | 1.03 (0.20, 5.44) | 0.971 |
| 25-36 months | - | - |  | 0.51 (0.11, 2.44) | 0.398 |  | 0.48 (0.07, 3.23) | 0.450 |
| ≥ 37 months | - | - |  | 1.29 (0.40, 4.20) | 0.675 |  | 1.73 (0.42, 7.12) | 0.445 |
| Major |  |  |  |  |  |  |  |  |
| Art | - | - |  | Reference |  |  | Reference |  |
| Science | - | - |  | 0.32 (0.13, 0.79) | 0.014 |  | 0.19 (0.06, 0.60) | 0.005 |
| Year of study |  |  |  |  |  |  |  |  |
| Year one | - | - |  | Reference |  |  | Reference |  |
| Year two | - | - |  | 1.47 (0.42, 5.21) | 0.551 |  | 0.41 (0.09, 1.77) | 0.232 |
| Year three | - | - |  | 1.84 (0.51, 6.63) | 0.349 |  | 0.90 (0.20, 4.15) | 0.892 |
| Year four | - | - |  | N/A |  |  | N/A |  |
| Grade Point Average |  |  |  |  |  |  |  |  |
| GPA < 2.50 | - | - |  | Reference |  |  | Reference |  |
| GPA 2.50-2.99 | - | - |  | 0.72 (0.25, 2.06) | 0.537 |  | 0.59 (0.16, 2.16) | 0.428 |
| GPA 3.00-3.49 | - | - |  | 2.23 (0.43, 3.42) | 0.699 |  | 2.19 (0.62, 7.71) | 0.222 |
| GPA 3.50-3.99 | - | - |  | 0.16 (0.02, 1.39) | 0.095 |  | 0.17 (0.01, 2.70) | 0.207 |
| GPA ≥ 4.00 | - | - |  | 0.51 (0.06, 4.73) | 0.554 |  | 1.09 (0.09, 12.55) | 0.948 |
| Hope | - | - |  | - | - |  | 1.55 (0.88, 2.74) | 0.130 |
| Flourishing | - | - |  | - | - |  | 0.91 (0.83, 1.00) | 0.051 |
| Purpose in life | - | - |  | - | - |  | 0.88 (0.75, 1.02) | 0.092 |
| Depression | - | - |  | - | - |  | 1.06 (0.98, 1.15) | 0.169 |
| Anxiety | - | - |  | - | - |  | 0.93 (0.86, 1.01) | 0.124 |
| Stress | - | - |  | - | - |  | 1.11 (1.01, 1.23) | 0.039 |
| Mindsets | - | - |  | - | - |  | 1.01 (0.53, 1.95) | 0.968 |
| Seeking help | - | - |  | - | - |  | 0.65 (0.28, 1.52) | 0.318 |

**Supplementary Table 3c** Logistic regression on suicidal behaviour among all participants

|  | Total (n=1,735) | | | | | | | |
| --- | --- | --- | --- | --- | --- | --- | --- | --- |
|  | Crude model | |  | Model 1 | |  | Model 2 | |
|  | OR (95% CI) | *P-value* |  | OR (95% CI) | *P-value* |  | OR (95% CI) | *P-value* |
| Social withdrawal |  |  |  |  |  |  |  |  |
| Yes | 2.29 (1.44, 3.63) | <0.001 |  | 2.35 (1.45, 3.81) | 0.001 |  | 0.74 (0.41, 1.34) | 0.318 |
| No | Reference |  |  | Reference |  |  | Reference |  |
| Type of hometown |  |  |  |  |  |  |  |  |
| City | - | - |  | Reference |  |  | Reference |  |
| Town | - | - |  | 0.74 (0.51, 1.09) | 0.132 |  | 0.70 (0.45, 1.07) | 0.097 |
| Village | - | - |  | 0.87 (0.59, 1.27) | 0.467 |  | 0.84 (0.55, 1.29) | 0.426 |
| Time of stay |  |  |  |  |  |  |  |  |
| ≤ 12 months | - | - |  | Reference |  |  | Reference |  |
| 13-24 months | - | - |  | 0.56 (0.29, 1.06) | 0.075 |  | 0.57 (0.27, 1.17) | 0.124 |
| 25-36 months | - | - |  | 1.04 (0.55, 1.94) | 0.913 |  | 1.23 (0.61, 2.48) | 0.555 |
| ≥ 37 months | - | - |  | 0.99 (0.60, 1.62) | 0.957 |  | 1.15 (0.66, 1.99) | 0.623 |
| Major |  |  |  |  |  |  |  |  |
| Art | - | - |  | Reference |  |  | Reference |  |
| Science | - | - |  | 0.99 (0.70, 1.40) | 0.950 |  | 1.37 (0.93, 2.03) | 0.111 |
| Year of study |  |  |  |  |  |  |  |  |
| Year one | - | - |  | Reference |  |  | Reference |  |
| Year two | - | - |  | 1.60 (0.92, 2.76) | 0.095 |  | 1.02 (0.55, 1.88) | 0.955 |
| Year three | - | - |  | 1.28 (0.74, 2.20) | 0.383 |  | 0.84 (0.45, 1.56) | 0.577 |
| Year four | - | - |  | 1.69 (0.88, 3.27) | 0.118 |  | 1.41 (0.68, 2.94) | 0.360 |
| Grade Point Average |  |  |  |  |  |  |  |  |
| GPA < 2.50 | - | - |  | Reference |  |  | Reference |  |
| GPA 2.50-2.99 | - | - |  | 0.63 (0.34, 1.14) | 0.126 |  | 0.72 (0.37, 1.42) | 0.345 |
| GPA 3.00-3.49 | - | - |  | 1.00 (0.57, 1.75) | 1.000 |  | 1.43 (0.76, 2.69) | 0.270 |
| GPA 3.50-3.99 | - | - |  | 0.81 (0.45, 1.48) | 0.499 |  | 1.57 (0.80, 3.09) | 0.191 |
| GPA ≥ 4.00 | - | - |  | 0.37 (0.14, 0.98) | 0.047 |  | 0.64 (0.22, 1.83) | 0.406 |
| Hope | - | - |  | - | - |  | 1.22 (0.98, 1.50) | 0.070 |
| Flourishing | - | - |  | - | - |  | 0.96 (0.92, 0.99) | 0.011 |
| Purpose in life | - | - |  | - | - |  | 0.96 (0.90, 1.02) | 0.148 |
| Depression | - | - |  | - | - |  | 1.09 (1.05, 1.21) | <0.001 |
| Anxiety | - | - |  | - | - |  | 1.01 (0.98, 1.05) | 0.454 |
| Stress | - | - |  | - | - |  | 1.00 (0.97, 1.04) | 0.893 |
| Mindsets | - | - |  | - | - |  | 1.38 (1.07, 1.77) | 0.013 |
| Seeking help | - | - |  | - | - |  | 0.92 (0.64, 1.32) | 0.645 |
